# Genome-Wide Identification and Expression Analysis of Aquaporin Gene Family in Tea Plant (*Camellia sinensis*)

**DOI:** 10.3390/plants14243786

**Published:** 2025-12-12

**Authors:** Huiyi Wang, Jiaojiao Tuo, Huixin Shao, Shiyi Chen, Hongli Cao, Chuan Yue

**Affiliations:** 1 Integrative Science Center of Germplasm Creation in Western China (Chongqing) Science City, College of Food Science, Southwest University, Chongqing 400715, China; wanghuiyi7777@163.com (H.W.); 18875503571@163.com (J.T.); s15937652172@163.com (H.S.); 18040445225@163.com (S.C.); lili9885@126.com (H.C.); 2 Chongqing Key Laboratory of Speciality Food Co-Built by Sichuan and Chongqing, Southwest University, Chongqing 400715, China

**Keywords:** tea plant, Aquaporins (AQPs), plasma membrane intrinsic proteins (PIPs), expression analysis, stress response, H_2_O_2_ transport

## Abstract

Aquaporins (AQPs) facilitate transmembrane transport of water and small solutes, critically influencing plant growth, development, and stress adaptation. However, tea plant AQPs (*CsAQPs*) remain incompletely characterized genome-wide. In this study, 61 *CsAQPs* were identified from the tea plant genome and could be classified into five subfamilies. The bioinformatics characteristics, including phylogenetic relationships, gene structures, chromosomal locations, conserved motifs, promoter *cis*-acting elements, and three-dimensional protein structure, were systematically investigated. Additionally, the expression patterns of *CsAQPs* in tea plants in response to abiotic and biotic stresses were comprehensively explored based on transcriptome data and qRT-PCR, suggesting that *CsAQPs* were closely associated with the tea plant responding to environmental adaptation. Notably, the functions of CsPIPs in response to drought and salt, as well as potential H_2_O_2_ transporters and their subcellular localization, were investigated in yeast. Collectively, our study delivers a complete genomic and evolutionary dissection of the *CsAQPs* gene family in the tea plant, providing valuable insights into their diverse functions for further investigation.

## 1. Introduction

Aquaporins (AQPs), a class of membrane-integrated channel proteins within the major intrinsic protein (MIP) family, mediate the transmembrane transport of water and small molecules such as glycerol, urea, CO_2_, and boric acid [1,2,3,4]. These proteins are ubiquitously distributed across diverse species, ranging from bacteria to higher eukaryotes [5]. In plants, AQPs are critical regulators of water homeostasis and stress adaptation, with their roles extending to the modulation of cellular responses to abiotic stressors. Based on subcellular localization and sequence homology, plant AQPs can be classified into five subfamilies: plasma membrane intrinsic proteins (PIPs), tonoplast intrinsic proteins (TIPs), nodulin26-like intrinsic proteins (NIPs), small basic intrinsic proteins (SIPs), and X intrinsic proteins (XIPs) [6]. Interestingly, XIPs are evolutionarily lost in monocots and in the *Brassicaceae* family of dicots [7,8,9], suggesting that the evolution of AQPs is complicated in plants. The number of AQPs varies among species, ranging from 35 members in *Arabidopsis thaliana* [8], 33 in *Oryza sativa* [10], and 86 in *Glycine max* [11]. Although the tea plant genome has been sequenced [12], genome-wide identification and functional characterization of *CsAQPs* remain insufficient; therefore, a comprehensive analysis of all members within the *CsAQPs* gene family is essential for fully elucidating their biological functions.

Structurally, AQPs share conserved features: cytoplasmic N- and C-termini, six transmembrane α-helices, and two asparagine-proline-alanine (NPA) motifs within loops B and E. These motifs form a three-dimensional “hourglass” structure within the lipid bilayer, essential for selective water permeation [13,14,15]. Another critical determinant of substrate specificity is the aromatic/arginine (Ar/R) filter, a narrow channel region composed of residues from transmembrane helices 2 and 5 (H2, H5) and loop E (LE1, LE2). The LE2 residue is highly conserved as arginine (R), further refining transport selectivity [16]. Statistical analysis of amino acid sequences of AQPs that can transport glycerol, known as aquaglyceroporins (GLPs), identified five highly conserved residues termed Froger’s positions: P1 in Loop C, P2 and P3 in Loop E, and P4 and P5 in TM6 [17].

Numerous studies have reported the pivotal role of the AQPs in regulating water and solute transport, which significantly influences plant growth, development, and stress responses [18]. In maize (*Zea mays*), overexpression of *PIP2;5* enhances root hydraulic conductance, highlighting AQPs’ direct role in water transport regulation [19]. The PIP subfamily in *Morus notabilis* plays a crucial role in responding to biotic infection [20]. Moreover, regulatory interactions between different AQP subfamilies can influence their subcellular localization and thereby modulate water permeability. [21]. Generally, AQP genes directly or indirectly participate in the regulation of various elements in cells, playing a key role in plant stress resistance [22].

The tea plant, a globally significant economic crop, thrives in subtropical climates with high humidity and precipitation [23]. The ongoing accumulation of greenhouse gases has induced global warming and consequent frequent climatic disasters, which have significantly impacted various agricultural sectors, including tea plant cultivation [24]. This environmental challenge has established stress resistance research as a pivotal area in tea plant studies. Previous investigations have identified several *CsAQPs* genes in tea plants from the transcriptome level, revealing their extensive involvement in responses to stresses, bud dormancy, and flower opening [25]. Additionally, several studies have shown that *CsAQPs* facilitate the tea plant’s response to selenium treatments and heat stress [26,27], reflecting that *CsAQPs* play an important role in tea plant growth and development, as well as stress response. However, the *CsAQPs* family at the genome-wide level and their functions remain largely unknown.

In this study, 61 *CsAQPs* genes were identified and characterized from the tea plant genome. Moreover, their phylogenetic relationships, gene structures, chromosomal locations, conserved motifs, gene duplication information, and regulatory *cis*-acting elements were comprehensively analyzed. The expression patterns of *CsAQPs* genes in different tissues during different seasons, in response to abiotic stresses of drought, cold, and salt treatments, and in response to biotic stresses of anthracnose and gray blight were investigated. The function and subcellular localization of CsPIPs were determined in the yeast system. Our results will provide valuable information for further functional studies of *CsAQPs* genes in the tea plant.

## 2. Results

### 2.1. Identification and Classification of AQP Genes in Tea Plant

BLAST searches were conducted against the tea plant genome using the known AQP protein sequences from *Arabidopsis thaliana*, *Vitis vinifera*, and *Vigna angularis as* queries. This led to the identification of 61 candidate *CsAQPs* (Table 1). These genes were classified into five subfamilies: 14 PIPs, 26 TIPs, 15 NIPs, 3 SIPs, and 3 XIPs, consistent with the nomenclature established in other plant species. Furthermore, each subfamily was subdivided into distinct subgroups: the NIPs formed seven subgroups (NIP1–NIP7), TIPs were categorized into five (TIP1–TIP5), PIPs were divided into PIP1 and PIP2, while SIPs and XIPs were each separated into two subgroups (SIP1/SIP2 and XIP1/XIP2, respectively).

The physicochemical properties of CsAQPs proteins were characterized, encompassing amino acid sequence length, molecular weight (MW), theoretical isoelectric point (pI), grand average of hydropathy (GRAVY), instability index (II), and aliphatic index (Table 1). Analysis showed CsAQPs exhibit lengths varying from 146 residues (CsNIP7;1) to 487 residues (CsNIP4;5), corresponding to MWs between 15.56 kDa and 52.71 kDa. Predicted pI values ranged from 4.78 to 10.44. Within the PIP, XIP, NIP, and SIP subfamilies, most members possess pI values exceeding seven, except for CsNIP4;5, CsNIP4;6, and CsNIP6;2. Conversely, 11 genes in the TIP subfamily have pI values greater than seven, while the remainder are below seven, indicating an acidic status for nearly half of the TIP subfamily. Instability indices were below 40 for most CsAQPs, signifying high relative stability in the tea plant; exceptions include CsNIP4;5 (42.96), CsNIP4;6 (41.18), and CsTIP2;3 (50.29). Aliphatic index values were calculated to be between 92.95 and 126.77. GRAVY values spanned +0.261 to +0.909, confirming the hydrophobic nature of these proteins. Transmembrane helix prediction revealed that most CsAQPs (39 proteins) possess six TMs, while CsNIP7;1, CsNIP7;2, CsTIP5;1 and CsTIP5;12 were predicted to contain only three TMs.

Subcellular localization predictions revealed diverse membrane associations for CsAQPs (Table 1). CsNIPs mainly occupied the plasma membrane; however, CsNIP1;1, CsNIP5;1, CsNIP5;2, and CsNIP6;2 exhibited dual localization (plasma membrane and vacuole), while CsNIP7;1 and CsNIP7;2 localized to the endoplasmic reticulum (ER). CsPIPs are primarily localized to the plasma membrane, whereas CsPIP2;5, CsPIP2;8, and CsPIP2;9 also showed vacuolar or ER localization. CsTIPs had complex distributions, occurring in the plasma membrane, vacuole, ER, and cytoplasm (cyto). CsXIPs are associated variably with the plasma membrane, ER, or vacuole. CsSIPs are localized exclusively to the plasma membrane and vacuole. This diversity highlights the broad functional roles of CsAQPs.

**Figure 1:**
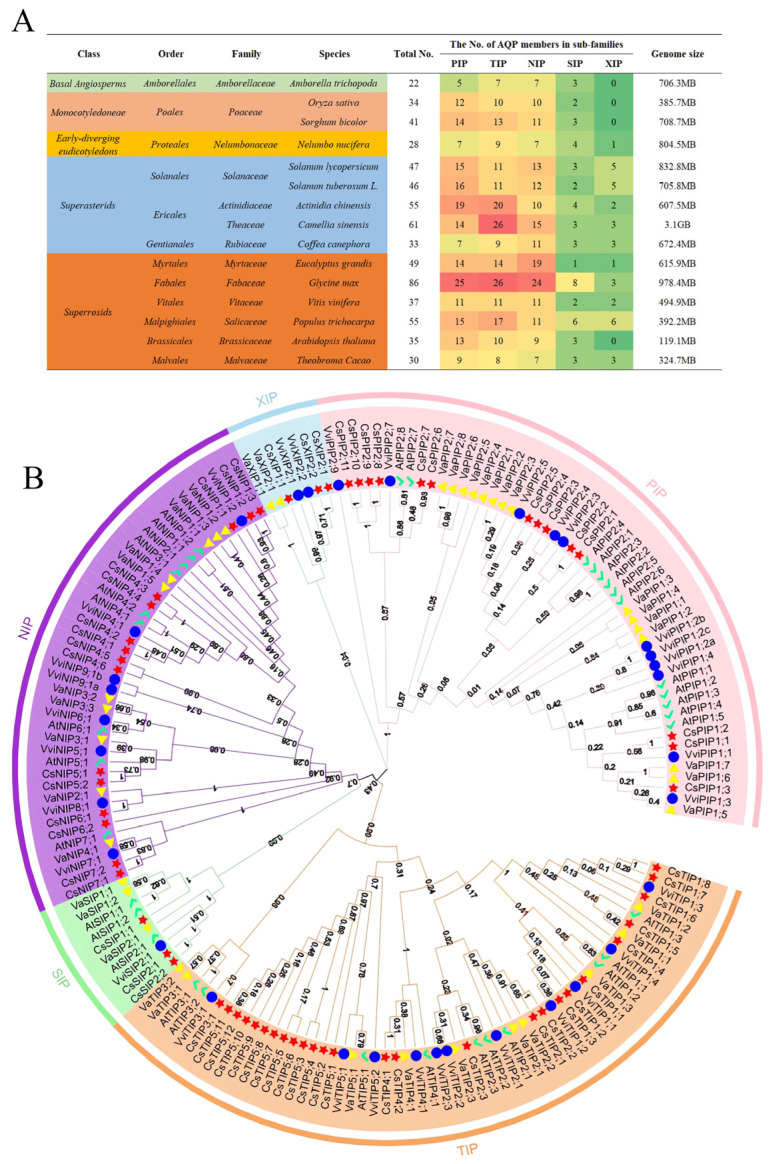
*AQP* family in different plant genomes and phylogenetic analysis of 61 AQP proteins from four species. (**A**) Distribution of genes belonging to the *AQP* family in different plant species genomes. Green background represents the plants that belong to Basal Angiosperms; faint-red background represents the plants that belong to Monocotyledoneae; yellow background represents the plants that belong to Early-diverging eudicotyledons; blue background represents the plants that belong to superasterids; orange background represents the plants that belong to superrosids. On the right side, the green-to-red color scale corresponds to the increasing gene numbers, as shown by the specific number. (**B**) Phylogenetic tree of the tea plant with other plant species. Red star represents the tea plant; yellow triangle represents *Vigna angularis*; green tick represents *Arabidopsis thaliana*; blue cycle represents *Vitis vinifera*.

To gain a full insight into AQP genes in plants, the AQP gene members involved in 14 species spanning *Basal Angiosperms*, *Monocotyledoneae*, *early-diverging eudicotyledons*, *Superasterids*, and *Superrosids* were used to analyze (Figure 1A). *Glycine max* contained the largest AQP gene complement (86 total genes: 25 PIPs, 26 TIPs, 24 NIPs, 8 SIPs, and 3 XIPs), followed by tea plant (61), *Actinidia chinensis* (55), and *Populus trichocarpa* (55). *Amborella trichopoda* (22) and *Nelumbo nucifera* (28) possessed fewer than 30 *AQP* genes. Analysis revealed no correlation between AQP gene number and genome size (Figure 1A); for example, *Vitis vinifera* (genome size 494.9 Mb) contains 37 *AQPs*, while *Amborella trichopoda* (706.3 Mb) contains only 22 *AQPs*. Within these plants, TIP, PIP, and/or NIP subfamilies predominated, while SIP and XIP subfamilies were comparatively scarce. Notably, no XIP genes were identified in *Amborella trichopoda*, *Oryza sativa*, *Sorghum bicolor*, or *Arabidopsis thaliana*. Phylogenetic analysis of AQPs from the tea plant, *Arabidopsis thaliana*, *Vitis vinifera*, and *Vigna angularis* elucidated evolutionary relationships (Figure 1B). The analysis clearly showed that the *AQPs* could be classified into five groups, and each group was clustered into different subgroups in the tree.

### 2.2. Synteny and Chromosomal Location Analysis of CsAQPs Gene Family Members

**Figure 2:**
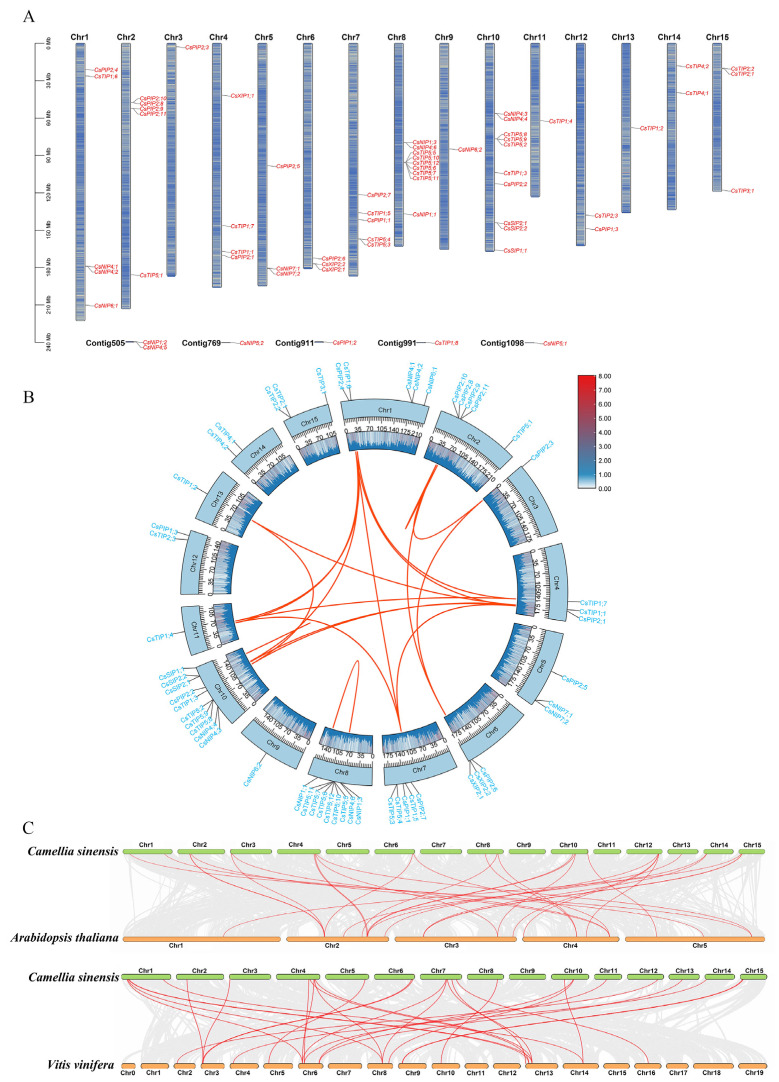
Chromosomal location analysis, intraspecific and interspecific collinearity analysis of *CsAQPs* gene family members. (**A**) Chromosomal location analysis of *CsAQPs*. (**B**) Intraspecific collinearity analysis of *CsAQP* genes. The red curves show the gene pairs that have undergone segmental duplication; (**C**) Collinearity analysis of *AQP* genes between the tea plant genome and *Arabidopsis thaliana*, and *Vitis vinifera* genomes. The gray lines in the background indicate the collinearity blocks between species. The collinear gene pairs are linked with red lines.

The chromosomal locations of the *CsAQPs* gene family members showed that these genes were unevenly distributed across the 15 chromosomes of the tea plant, with six genes located on unassembled contigs (Table 1, Figure 2A). Chr-10 contained the highest number of *CsAQPs* genes, whereas Chr-3, Chr-9, Chr-11, and Chr-13 each harbored only one gene. The different subfamilies of *CsAQPs* genes were spread across various chromosomes. To explore the evolutionary mechanisms underlying the expansion of the *CsAQPs*, we performed an intraspecies collinearity analysis. In total, 16 syntenic gene pairs were identified, indicating that 18 *CsAQPs* originated from gene duplication events (Figure 2B; Table S1). These results suggest that whole-genome segmental duplication played a major role in the expansion of the *CsAQPs*. The Ka/Ks ratios for all 16 duplicated gene pairs were less than 1 (Table S2), ranging from 0.0391 (*CsTIP1;6*/*CsTIP1;4*) to 0.3344 (*CsPIP2;9*/*CsPIP2;10*), showingthat they have undergone purifying selection. In addition to intraspecific collinearity, we also performed interspecific collinearity analysis among the tea plant, *Arabidopsis thaliana*, and *Vitis vinifera* (Figure 2C). A total of 17 syntenic gene pairs were detected between the tea plant and *Arabidopsis thaliana*, and 21 were shared with *Vitis vinifera*. Notably, 12 *CsAQPs* were conserved in syntenic blocks across species, including *CsNIP4;1*, *CsPIP2;3*, *CsPIP2;6*, and *CsTIP1;2*, suggesting that these orthologous pairs may have important functional or evolutionary roles.

### 2.3. Analysis of Gene Structure, Conserved Domains, and Motifs of CsAQPs Gene Family Members

**Figure 3:**
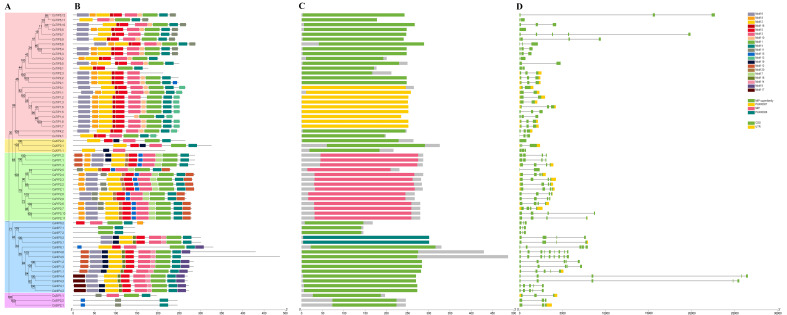
Visualization of *CsAQPs* conserved domains in the tea plant. (**A**) The phylogenetic tree was constructed, TIP was covered by red, XIP was covered by yellow, PIP was covered by green, NIP was covered by blue, and SIP was covered by purple. (**B**) Conserved motif composition of CsAQP proteins. The motifs were colored with different colors. (**C**) Representation of different conserved protein domain families. (**D**) Gene structure analysis of *CsAQPs* genes.

To gain insights into the structural evolution and functional divergence of the *CsAQPs* gene family, the gene structures and conserved protein motifs were analyzed. Based on phylogenetic relationships, the 61 CsAQPs proteins were classified into five evolutionary branches, all exhibiting highly conserved motif patterns and domain architectures (Figure 3 and Figure S1). Twenty conserved motifs were identified (Figure S2). Most AQP proteins contained 10 motifs, while CsNIP7;1 and CsNIP7;2 had only two motifs each (Figure 3B). Generally, the SIPs have fewer conserved domains [28,29]; in this study, we found that CsSIPs had 3–4 motifs, with shared motifs 4 and 11, and motifs 1 and 3 were located near the C-terminus in CsSIP1s, whereas motif 15 was located at the N-terminus in CsSIP2s. Motif 1 and motif 2 correspond to the two NPA motifs located at LE and LB, which are closely related to the water transport activity of AQPs. CsTIP2;3, CsXIP1;1, CsSIP2;1 and CsSIP2;2 lack motif 1, while CsNIP6;2, CsNIP7;1, CsNIP7;2, and CsSIPs lack motif 2. Motif 6, which contains the highly conserved AEFxxT sequence in TM1 [3], was absent in CsSIPs, CsXIPs, CsTIP5;1, CsTIP5;2, CsTIP4;1, CsPIP2;5, CsNIP6;1, CsNIP6;2, CsNIP7;1, and CsNIP7;2. Exon–intron structure analysis revealed that the number of exons in *CsAQPs* ranged from two to nine (Figure 3D). Genes within the same clade showed similar exon–intron architectures, both in number and length. These findings suggest that the conserved gene structures and motif compositions of CsAQPs reflect their evolutionary relationships and are closely associated with functional similarities.

### 2.4. Protein Structures and Substrate-Specific Residues of CsAQPs

**Figure 4:**
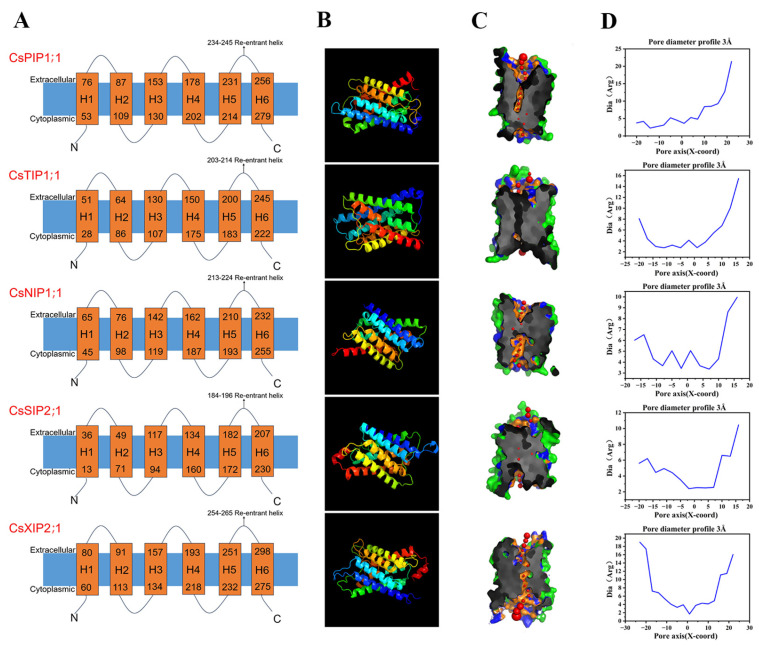
Three-dimensional (3D) model prediction and pore analysis of five representative CsAQPs. (**A**) Transmembrane structure; (**B**) 3D structure of CsPIP1;1, CsTIP1;1, CsNIP1;1, CsSIP2;1 and CsXIP2;1. Models were constructed using the Phyre2 server; (**C**) The pore morphology; (**D**) Dimensions of CsAQPs proteins were analyzed by the PoreWalker software, which provides a cross-sectional view and pore size.

In order to phylogenetically differentiate the five AQP subfamilies, the representative proteins of CsPIP1;1, CsTIP1;1, CsNIP1;1, CsSIP2;1, and CsXIP2;1 were selected for structural homology modeling prediction (Figure 4). Despite their distinct evolutionary lineages, most of the CsAQPs contained six transmembrane helices (Table 1). The tertiary structures of five representative CsAQPs were conserved, each featuring six transmembrane helices (TM1–TM6) that form an hourglass-shaped pore, a hallmark topology of the MIP superfamily. These helices are interconnected by five helical loops (HB1–HB5), together constituting a narrow aqueous channel traversing the membrane (Figure 4A). Two NPA motifs embedded in opposing half-helices converge at the pore center, generating a steric constriction point (Figure 4B). Pore radius calculations further revealed that the transmembrane helices form a constricted channel neck across the membrane (Figure 4C). The minimum diameters of these water channels ranged from 1.69 to 3.35 Å among the different isoforms (Figure 4D), which is critical for selective water permeation in AQPs.

AQPs are primarily recognized for their role in transporting small molecules. Specific amino acid domains and residues within AQPs determine their selectivity in transporting substrates. Particularly, the two NPA motifs, the Ar/R filter, and Froger’s positions are crucial for the physiological functions of AQPs. The Ar/R filter was located on the extracellular side, where it contributes to substrate selectivity. Also, conserved R can form hydrogen bonds with H_2_O and repel H^+^ to maintain the proton potential gradient [15]. The Froger’s position consists of five amino acid residues, which can be used as a basis for screening AQPs with the ability to transport water and glycerol [17]. In this study, these key amino acid residues were investigated (Table 2). Interestingly, in CsPIP1-1, LB and LE were both NPA, the Ar/R filter was composed of FHTR, and Froger’s positions were composed of QSAFW. CsTIP1;1, LB, and LE were all NPA, and the Ar/R filter was composed of HIAV; Froger’s positions were composed of TSAYW. LB and LE in CsNIP1;1 were both NPA, and the Ar/R filter was composed of this WVAR; Froger’s positions were composed of FSAVL; in CsSIP2;1, LB and LE were NPL and NPA, LB alanine (A) was converted to leucine (L), and Ar/R filter was composed of S-GS, Froger’s positions was composed of FVAYW; in CsXIP2;1, LB and LE were NPI and NPA, LB alanine (A) was converted to isoleucine (I), and Ar/R filter was composed of IVAR, Froger’s positions was composed of VCAFW.

### 2.5. Protein–Protein Interaction Network of CsAQPs

Protein–protein interaction networks for CsAQPs were predicted using *Arabidopsis thaliana* orthologs (Figure S3). Analysis revealed orthologous relationships for 20 CsAQPs; five additional proteins interacted with multiple CsAQPs. CsPIPs displayed the strongest intra-subfamily interactions. Specific CsPIPs, including CsPIP2;3, CsPIP1;2, CsPIP1;3, CsPIP2;5, and CsPIP2;11, showed strong associations, indicating functional coordination between CsPIP1 and CsPIP2 subgroups. Strong interactions were also characterized within CsXIPs and CsTIPs. Moreover, we found that several CsAQPs were predicted to interact with the RING-type zinc finger protein NEP1-interacting protein 2 (NIP2). Members of PIP, TIP, NIP, and XIP subfamilies were connected to NAC domain-containing protein 91 (NAC091), a transcription activator essential for virus basal resistance [30]. Several AQP subfamily members were predicted to interact with PAMP-induced secreted peptide 1 (PIP1), an endogenous immune elicitor and defense regulator. This network analysis suggests that protein interactions may play a critical role in regulating AQP activity.

### 2.6. Analysis of Cis-Acting Elements in CsAQPs

**Figure 5:**
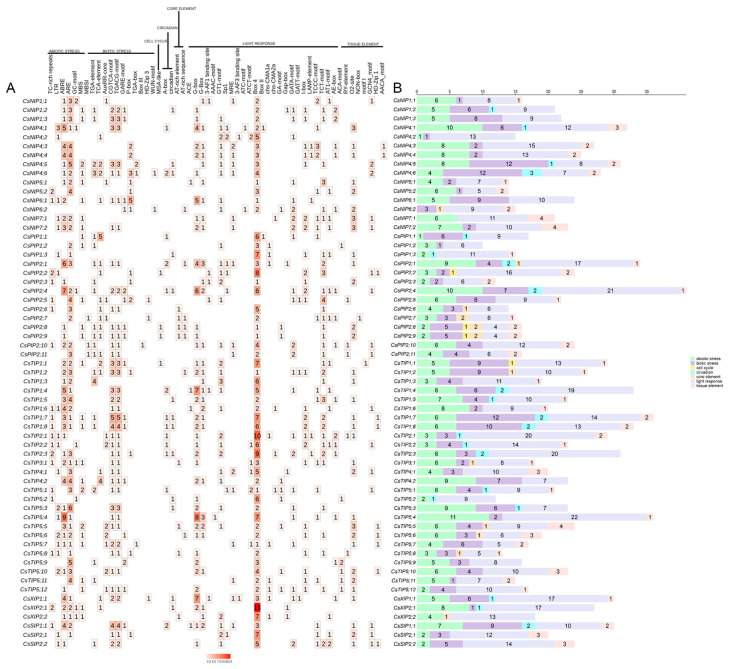
Analysis of *cis*-acting element numbers in *CsAQPs* genes. (**A**) The different colors and numbers of the grid indicated the numbers of different promoter elements in *CsAQPs* genes. (**B**) The different colored histograms represented the sum of the *cis*-acting elements in each category.

In this study, cis-regulatory elements within 2000 bp promoter regions were analyzed to delineate transcriptional control mechanisms of *CsAQPs* expression in the tea plant. These cis-acting elements were categorized into seven classes: abiotic stress elements, biotic stress elements, cell cycle elements, circadian elements, core elements, light response elements, and tissue elements (Figure 5; Table S3). In total, 26 types of light-responsive elements, such as AAAC-MOTIF, I-Box, and LAMP elements, were predicted within the *CsAQPs* promoter regions. In addition, numerous stress-related elements, such as AREs, CGTCA motifs, and ABRE, were widely distributed among the *CsAQPs* members in the tea plant. Specifically, *CsTIP5;4* contained nine ABRE elements, *CsTIP5;3* had six ARE elements, and *CsTIP1;7* had five CGTCA motifs. Moreover, certain elements involved in plant tissue-specific expression, like O_2_-sites, HD-Zip1, and NON-box, were identified in the promoters of *CsAQPs*, and more than half of the *CsAQPs* (32) contained at least one O_2_-sites on promoters, suggesting that *CsAQPs* genes play a significant role in tea plant growth and development. Additionally, circadian-related elements (e.g., MSA-like motifs) were also predicted in several genes.

### 2.7. Analysis of Tissue-Specific Expression of CsAQPs in Tea Plant

**Figure 6:**
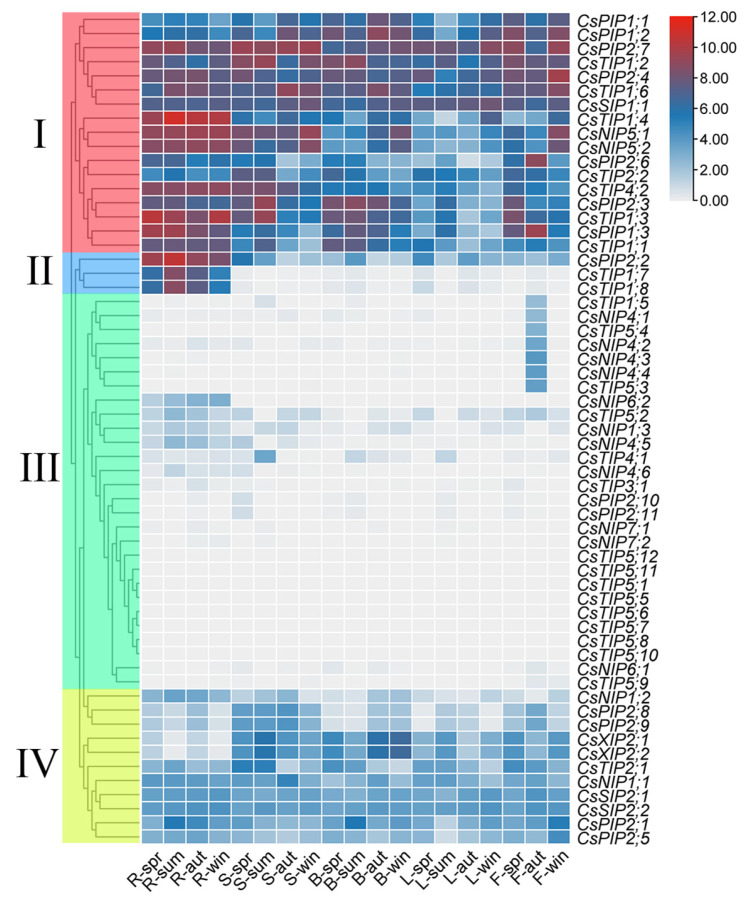
Expression analysis of *CsAQPs* in different tissues in different seasons. The red and blue colors represent high and low expression levels, respectively. R: root; S: stem; B: bud; L: leaf; F: flower; spr: spring; sum: summer; aut: autumn; win: winter.Group I comprises the genes from *CsPIP1;1 *to* CsTIP1;1; *Group II, from* CsPIP2;2 *to* CsTIP1;8;* Group III, from* CsTIP1;5 *to* CsTIP5;9;* and Group IV, from* CsNIP1;2 *to* CsPIP2;5.*

Expression patterns of *CsAQPs* across root, stem, bud, leaf, and flower tissues were analyzed using the transcriptomic dataset PRJEB39502, revealing four expression clusters (Figure 6). Group I genes showed detectable transcription in all tissues, with maximal accumulation in roots (*CsTIP1;4*, *CsPIP1;3*, and *CsNIP5;1*). Group II exhibited strictly root-specific expression (*CsPIP2;2*, *CsTIP1;7*, and *CsTIP1;8*) and minimal transcription elsewhere. Group III demonstrated the lowest overall expression, although six genes (*CsNIP*4*;1*, *CsNIP4;2*, *CsNIP4;3*, *CsNIP4;4*, *CsTIP1;5*, and *CsTIP5;4*) activated exclusively in autumn floral tissues, suggesting floral regulation roles. Group IV maintained moderate ubiquitous expression, with elevated accumulation in buds and stems (*CsXIP2;1* and *CsXIP2;2*). Seasonal analysis further identified dynamic expression patterns: root, stem, and leaf tissues showed expression peaks in summer or winter (*CsTIP1;3*, *CsTIP1;4*, *CsPIP2;2*, *CsPIP2;3*). In buds, two divergent patterns were observed: elevated expression in spring/summer (*CsPIP2;3*, *CsTIP1;2*, *CsPIP2;4*) versus peaks in autumn/winter (*CsXIP*2;1, *CsNIP*5;1, *CsTIP*1*;6*). Floral tissues exhibited either spring/winter maxima (*CsPIP2;4*, *CsPIP2;7*) or autumn-specific upregulation (*CsPIP1;3*, *CsPIP2;6*).

### 2.8. Expression Analysis of CsAQPs in Tea Plant Response to Abiotic Stresses

**Figure 7:**
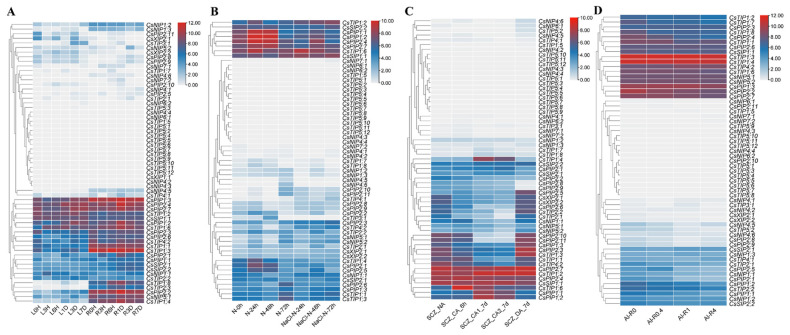
Expression analysis of *CsAQPs* in different abiotic stresses and Al treatments. (**A**) Expression analysis of *CsAQPs* in response to drought treatments. The clustering was performed based on the log2 fold change for each gene in a comparison between the stressed and control conditions. L: leaf, R: root, H: hour, D: day. Drought stress imposed via 25% polyethylene glycol (PEG) 6000; (**B**) Expression analysis of *CsAQPs* in response to salinity treatments. The clustering was performed based on the log2 fold change for each gene in a comparison between the stressed and control conditions. N: control conditions, NaCl: salt stress imposed via 200 mM NaCl; (**C**) Expression analysis of *CsAQPs* in response to cold stress. NA (Unacclimated, growing at 25 °C), CA6 h (Fully acclimated, growing at 10 °C for 6 h), CA1–7 d (Domesticated, growing at 10/4 °C at day/night for 7 days), CA2–7 d (Domesticated, growing at 4/0 °C at day/night for 7 days) and DA-7 d (Recovering at 25 °C for 7 days); (**D**) Expression analysis of *CsAQPs* in response to different Al doses (0, 0.4, 1 and 4 mmol L^−1^). The clustering was performed based on the log2 fold change for each gene in a comparison between the stressed and control conditions.

Abiotic stress frequently impacts tea plant growth stages, ultimately reducing yield and quality. To elucidate *CsAQPs* genes’ roles in abiotic stress responses, in this study, their expression patterns under drought, salinity, cold, and aluminum treatments were investigated. Transcriptome data from drought-stressed plants, with leaves and roots sampled at 0, 3 h, 6 h, 1 d, 3 d, and 7 d for RNA-Seq, were analyzed. As shown in Figure 7A, most *CsAQPs* in leaves were significantly downregulated during the first 6 h of drought stress, but their expression increased as the treatment was prolonged to 3 d and 7 d. For instance, the expression of *CsPIP1;2*, *CsPIP1;3*, *CsPIP2;4*, and *CsPIP2;7* decreased markedly at 3 h and/or 6 h but was substantially induced at 3 d and/or 7 d. In contrast, the expression of *CsNIP1;1*, *CsPIP2;8*, *CsPIP2;9*, *CsPIP2;11*, *CsTIP1;1*, *CsTIP1;3*, and *CsXIP2;1* remained suppressed in leaves under drought, whereas *CsTIP1;6* and *CsTIP1;4* were upregulated with prolonged stress. A similar trend was observed in roots, where most genes were downregulated early but upregulated after 1 d. Specifically, genes such as *CsNIP1;3*, *CsPIP2;6*, *CsTIP1;1*, and *CsTIP4;2* were downregulated, while *CsPIP1;2*, *CsPIP2;1*, *CsPIP2;2*, *CsPIP*2;4, *CsTIP1;4*, *CsTIP1;6*, *CsTIP1;8*, *CsTIP2;2*, *CsTIP2;3*, and *CsSIP2;1* were induced. Furthermore, changes in *CsAQPs* expression were generally more pronounced in roots than in leaves (Figure 7A).

Salt stress induced downregulation in most *CsAQPs* compared to unstressed controls (Figure 7B). Nearly all *CsPIPs* and *CsTIPs* members decreased under salt stress. Notably, *CsNIP1;1*, *CsNIP5;1*, *CsNIP5;2*, *CsTIP1;2*, *CsTIP4;1*, *CsTIP4;2*, *CsXIP2;1*, and *CsSIP1;1* exhibited upregulated expression during salt treatment, suggesting potential involvement in tea plant salt resistance.

Transcriptome data from cold treatments in five phases—control, acute cold, CA1-7 d, CA2-7 d, and recovery—revealed CsAQPs responses in tea plants (Figure 7C). Most genes showed repression during cold exposure but increased after recovery. Conversely, *CsPIP1;1*, *CsPIP1;2*, *CsTIP1;2*, *CsTIP1;4*, *CsTIP1;6*, *CsSIP2;1*, and *CsSIP2;2* exhibited significant induction, suggesting cold stress involvement. Given the known aluminum tolerance of tea plants, we also analyzed *CsAQPs* expression under Al treatments (Figure 7D). Increasing aluminum concentrations downregulated 19 genes and upregulated 5 genes. Specifically, *CsNIP1;1*, *CsPIP2;2, CsTIP1;4*, and *CsTIP1;6* expression decreased progressively with higher Al^3+^ doses, while *CsNIP4;1*, *CsTIP4;1*, *CsTIP4;2*, *CsPIP2;7*, and *CsSIP1;1* increased. Most genes remained unaffected by aluminum exposure. These findings identify key candidate genes potentially involved in regulating tea plant responses to abiotic stress.

### 2.9. Expression Analysis of CsAQPs in Tea Plant Response to Biotic Stresses

**Figure 8:**
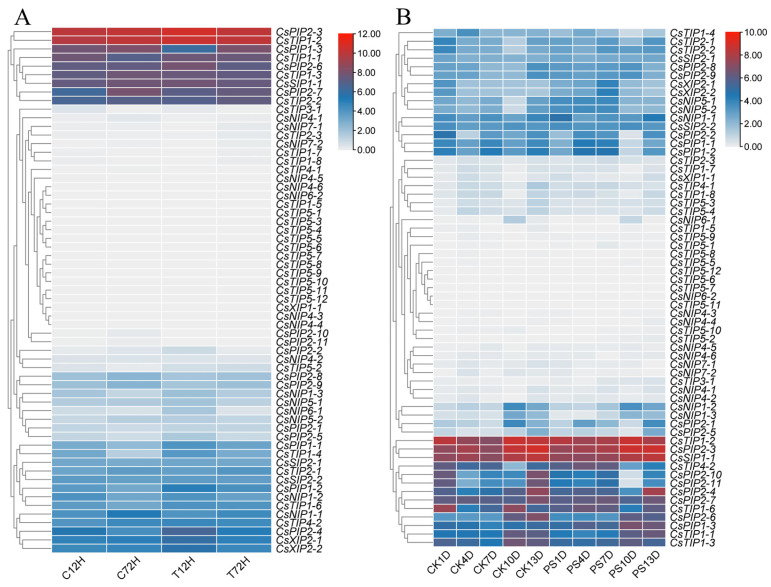
Expression analysis of *CsAQPs* in different biotic stresses. (**A**) Expression analysis of *CsAQPs* in response to anthracnose. The clustering was performed based on the log2 fold change for each gene in a comparison between the stressed and control conditions. C represents inoculation with sterile water; T represents the tea leaves after *Colletotrichum camelliae* inoculation; (**B**) Expression analysis of *CsAQPs* in response to gray blight. The clustering was performed based on the log2 fold change for each gene in a comparison between the stressed and control conditions. CK represents inoculation with sterile water; PS represents inoculation with the spore suspension of *Pseudopestalotiopsis* sp. at a concentration of 10^7^ conidia/mL.

Anthracnose (infected by *Colletotrichum camelliae*) and gray blight (infected by *Pestalotiopsis theae* (Sawada) Steyaert) are major fungal diseases that severely impact tea plant growth [31,32]. It has been well recognized that *AQP* genes play a crucial role in plant response to disease resistance. In this study, we investigated the expression patterns of *CsAQPs* following infection with these pathogens. As shown in Figure 8, the expression of most genes did not change significantly compared with healthy controls. However, after anthracnose infection, *CsPIP1;1*, *CsPIP1;2*, *CsPIP2;1*, *CsPIP2;3*, *CsPIP2;4*, *CsTIP1;1*, *CsTIP1;4*, *CsTIP1;6*, and *CsTIP2;2* expressions were upregulated, while *CsNIP1;1* and *CsPIP1;3* were downregulated (Figure 8A), suggesting their potential involvement in the response to anthracnose. Upon gray blight infection, the transcription levels of *CsNIP5;1*, *CsNIP5;2*, *CsPIP1;3, CsPIP2;3*, *CsPIP2;4*, *CsPIP2;8*, *CsTIP2;2,* and *CsXIP2;1* were upregulated, whereas *CsPIP1;1* and *CsPIP2;10* were downregulated (Figure 8B). These results indicate that certain *CsAQPs*, especially specific *CsPIPs* members, are involved in the tea plant’s defense responses against fungal pathogens.

### 2.10. Expression Analysis of CsPIPs in Tea Plant Response to Abiotic Stresses by qRT-PCR

**Figure 9:**
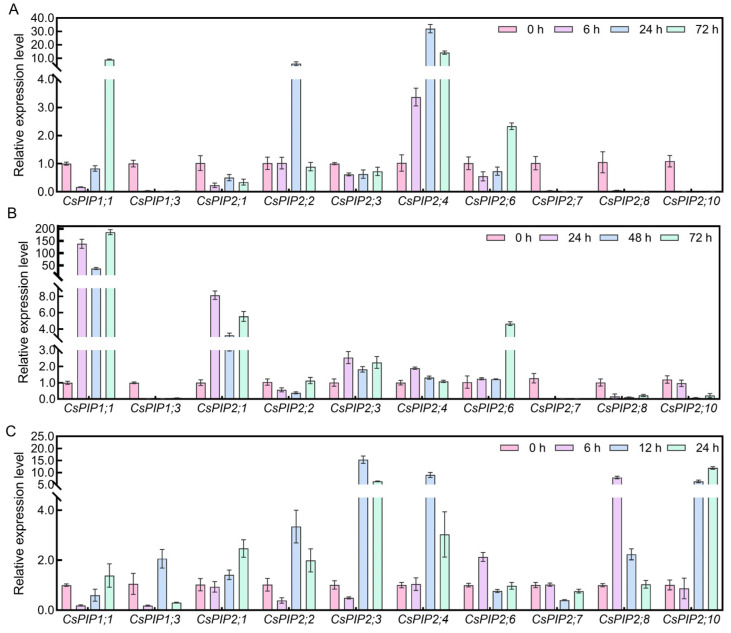
Expression analysis of *CsPIPs* in response to abiotic stress by using qRT-PCR detection (**A**) Relative expression level of *CsPIPs* in response to drought stress. (**B**) Relative expression level of *CsPIPs* in response to salt stress. (**C**) Relative expression level of *CsPIPs* in response to cold stress.

In this study, the expression patterns of ten *CsPIPs* were selected and determined using qRT-PCR. As shown in Figure 9, under drought stress, the expression levels of most *CsPIPs* exhibited downregulation patterns, especially *CsPIP1;3*, *CsPIP2;7*, *CsPIP2;8*, and *CsPIP2;9* were significantly repressed; whereas the expression levels of *CsPIP1;1*, *CsPIP2;2*, and *CsPIP2;4* were upregulated. Moreover, the expression levels of *CsPIP1;1*, *CsPIP2;1*, *CsPIP2;3*, and *CsPIP2;6* were dramatically induced by salt treatment; the transcription of *CsPIP1;3*, *CsPIP2;7*, *CsPIP2;8*, and *CsPIP2;10* was totally repressed under salt stress. Interestingly, most of the genes were upregulated by cold treatment, especially *CsPIP2;1*, *CsPIP2;2*, *CsPIP2;3*, *CsPIP2;4*, *CsPIP2;8*, and *CsPIP2;10*.

### 2.11. Functional Analysis of CsPIPs in the Yeast System

**Figure 10:**
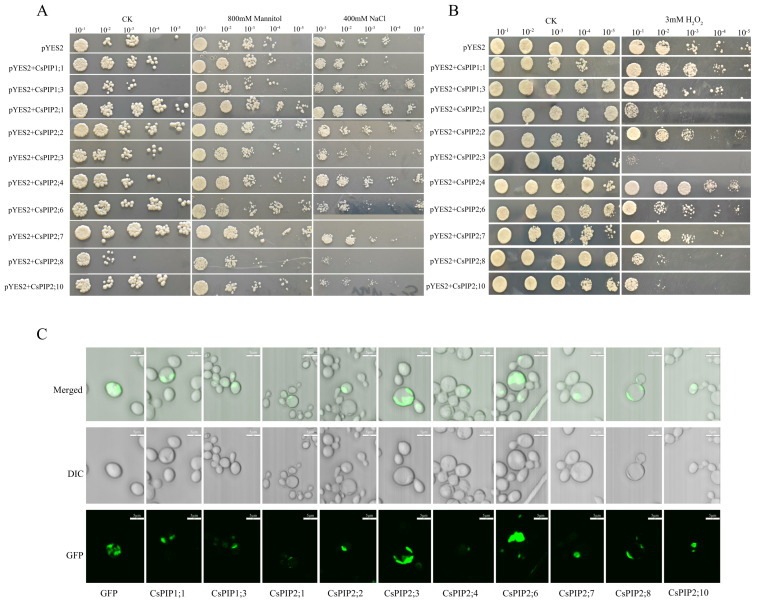
Functional analysis of CsPIPs in transgenic yeast. *CsPIPs* were constructed into the pYES2-GFP and transformed into the yeast strain NMY51. (**A**) Diluted yeast cells were spotted on SD-URA plates with 400 mM NaCl, 800 mM mannitol. (**B**) Diluted yeast cells were spotted on SD-URA plates with 3 mM H_2_O_2_. (**C**) The subcellular localization of CsPIPs in yeast cells.

The yeast expression system serves as a valuable tool for investigating protein function. Indeed, this system has proven instrumental in elucidating the biological roles of key proteins in eukaryotes [33,34]. To further investigate the functions of *CsPIPs*, ten *CsPIPs* were cloned into the pYES2-GFP vector and transformed into the yeast strain NMY51. In spot assays under NaCl stress, yeast expressing *CsPIP2;8* and *CsPIP2;10* showed growth sensitivity, whereas strains expressing *CsPIP1;3*, *CsPIP2;1*, *CsPIP2;2*, and *CsPIP2;4* exhibited high tolerance (Figure 10A). Under drought stress simulated using a medium containing 800 mM mannitol, yeast expressing *CsPIP2;1*, *CsPIP2;2*, *CsPIP2;6*, and *CsPIP2;7* formed larger colonies than the control, while the growth of yeast carrying *CsPIP1;3, CsPIP2;3*, *and CsPIP2;8* was inhibited (Figure 10A). Given the reported role of PIPs in H_2_O_2_ transport, we also assessed the H_2_O_2_ transport capability of *CsPIPs*. Among the transgenic yeast strains, the growth of those expressing *CsPIP2;1*, *CsPIP2;3*, *CsPIP2;6*, *CsPIP2;8*, and *CsPIP2;10* was significantly inhibited under H_2_O_2_ stress. In particular, *CsPIP2;3* expressing yeast showed high sensitivity to H_2_O_2_, suggesting that these *CsPIPs* may facilitate H_2_O_2_ transport (Figure 10B).

Moreover, the subcellular localization of CsPIPs was investigated in yeast cells. As shown in Figure 10C, the GFP fluorescent protein signals of CsPIPs were primarily observed to be in the plasma membrane, aligning with the results of subcellular localization prediction (Table 1).

## 3. Discussion

AQPs are crucial transmembrane channels that facilitate the transport of water and small solutes in plants. While a subset of 20 *CsAQPs* had been previously identified in the tea plant [25], a comprehensive genomic overview was lacking. Our genome-wide analysis now reveals a notable expansion of this family in the tea plant, with 61 identified *CsAQP* genes. This number exceeds that of many other species (Figure 1A), a phenomenon potentially linked to the Polemonioids–Primuloids-core Ericales (PPC) whole-genome duplication event, which may have amplified genetic material pertinent to stress adaptation and specialized metabolism [35]. This expansion appears to have been driven by various duplication events, with purifying selection (Ka/Ks < 1) playing a major role in shaping the family’s evolution, suggesting strong functional constraints on most members. Furthermore, the substantial collinearity observed with species like *Vitis vinifera* underscores deep evolutionary conservation, while the unique expansions highlight lineage-specific adaptations in the tea plant.

The composition of the CsAQP family, featuring five subfamilies (PIPs, TIPs, NIPs, SIPs, and XIPs) with a notable expansion of CsTIPs, aligns with patterns observed in other perennial plants like *Actinidia chinensis, Glycine max,* and *Populus trichocarpa* (Figure 1A) but contrasts with the dominance of PIPs in cucumber [36] or NIPs in sugar beet [28]. Interestingly, three *CsSIPs* and three *CsXIPs* genes were also identified from the tea plant, and each of them could be divided into two groups. Gene structures and protein motifs exhibited high conservation within subfamilies. This structural diversity with sequence conservation may reflect functional heterogeneity among isoforms. The functional differentiation among subfamilies, with PIPs and TIPs governing the transport of water and key signaling molecules like H_2_O_2_ and CO_2_ [1], NIPs facilitating the uptake of beneficial metalloids like silicic and boric acid, and SIPs/XIPs handling substrates such as glycerol and urea [37]—collectively equips the tea plant to precisely manage a wide array of solutes. This functional diversity, stemming from gene family expansion and divergence, likely constitutes a critical molecular foundation for the tea plant’s adaptation to diverse environmental challenges.

Such functional diversity likely supports environmental adaptation during speciation. For instance, it has been reported that the domesticated olive (*Olea europaea*) contains more *AQP* genes than its wild relatives [38]. Therefore, *CsAQPs* gene expansion represents a critical mechanism for evolutionary adaptation in tea plants. In this study, we also investigated the potential CsPIPs in H_2_O_2_ transportation using yeast cells and identified *CsPIP2;1*, *CsPIP2;3*, *CsPIP2;6*, *CsPIP2;8*, and *CsPIP2;10* as having a high ability to transporting H_2_O_2_ (Figure 10B), suggesting that these genes may play important roles in tea plant response to stress by modulating H_2_O_2_ transportation.

The molecular architecture of aquaporins is evolutionarily conserved across plants, and tea plant CsAQPs are no exception. Most CsAQPs retain the characteristic six transmembrane α-helices that form the aqueous pore, with cytoplasmic N- and C-termini. Critically, all identified CsAQPs contain at least one conserved NPA (Asn-Pro-Ala) motif (Figures S1 and S2). The NPA constriction governs substrate size permeability, while Ar/R filters and P1-P5 residues determine substrate specificity [15,39]. CsAQPs display 17 distinct Ar/R filter types (Table 2), exceeding the nine types in *Arabidopsis thaliana*’s 35 AQPs, indicating greater substrate transport diversity. For instance, CsNIP1;2 shares the WVAR Ar/R filter with AtNIP1;2 [40] and *Prunus avium* NIP1;1 [41], suggesting conserved transport functions. CsPIPs contain water-transporting FHTR filters, documented to transport CO_2_, boric acid, urea, and H_2_O_2_ in other species [42]. CsTIP1s (HIAV filter) and CsTIP2s (HIGR filter) resemble orthologs, indicating potential H_2_O_2_ or urea/ammonia transport roles [43]. Pore feature prediction revealed channel diameters of 1.69–3.35 Å, enabling broad substrate permeability that supports functional diversity. Protein–protein interaction predictions showed that AQPs had strong interactions with each other, particularly between members of the CsPIP1 and CsPIP2 subfamilies, indicating that the formation of hetero- or homotetrameric complexes is a key regulatory mechanism for trafficking and function [44]. Furthermore, several CsAQPs were also predicted to interact with proteins such as the NAC091 transcription factor, PIP1, and NEP1-interacting protein 2 (Figure S3), suggesting potential roles in biotic stress responses. Similar functional interactions have been reported in papaya, where TIPs interact with PAT proteins and NIPs bind to ACR proteins [45]. Together, these findings implicate CsAQPs in a wide range of physiological processes in the tea plant.

In tea plants, CsAQPs were predicted to have a high possibility of being located in the plasma membrane, vacuole, and endoplasmic reticulum, indicating their diverse functions in plants. In this study, we determined the subcellular localization of CsPIPs in yeast, showing they were mainly located in the plasma membrane (Figure 10C). Except for their subcellular localization diversity, *CsAQPs* exhibit tissue-specific expression patterns in tea plants. Most expressed genes showed elevated transcript levels in roots, indicating fundamental roles in water diffusion or root regulatory functions. In support of a role in root development, *CsTIPs* demonstrated particularly high root abundance. Reinhardt et al. indicated that tonoplast-localized *AtTIP1;1*, *AtTIP1;2*, and *AtTIP2;1* facilitate lateral root formation in *Arabidopsis thaliana* [46]. Conversely, *CsPIP2;7*, *CsPIP2;4*, *CsPIP2;6*, and *CsPIP1;3* displayed higher floral expression, implying a potential role in flowering regulation. This is consistent with studies in rose and other species, where PIP genes have been critically implicated in flowering control [47,48,49,50]. Genes including *CsPIP2;3*, *CsTIP1;2*, and *CsTIP1;6* exhibited increased bud expression, aligning with prior evidence of their functions in bud dormancy cycles [25]. Interestingly, the expression patterns of several genes varied with seasonal changes across different tissues, indicating that these genes may play critical roles in the tea plant’s adaptation to environmental fluctuations.

The presence of diverse abiotic and biotic stress-responsive cis-acting elements in the promoters of *CsAQPs* suggests their central role in the tea plant’s transcriptional reprogramming upon stress perception. Our expression profiling under various stress conditions revealed a complex regulatory landscape, characterized by a predominant downregulation of most *CsAQPs*. This widespread suppression may represent a conserved strategy to minimize water and solute loss by reducing membrane permeability under adverse conditions, a phenomenon observed in tea plants [25] and other species [28,40,51,52]. In contrast, the significant upregulation of a subset of *CsAQPs* points to their specialized roles in stress adaptation. These distinct expression profiles suggest that *CsAQPs* play critical roles in maintaining cellular water homeostasis during adverse conditions. Growing evidence indicates that AQPs facilitate H_2_O_2_ transport, which is involved in stress signaling and growth regulation. For instance, in melon, *CmPIP2;3* enhances cold tolerance via CmABF2/3-mediated H_2_O_2_ transport [53]. Similarly, *OsPIP2;2* in rice regulates innate immunity through H_2_O_2_ signaling [54] and in *Pyrus betulaefolia*, a *PbERF3–PbHsfC1a–PbNCED4–PbPIP1;4* regulatory module mediates drought adaptation via H_2_O_2_ transport [55]. In this study, we detected the expression levels of *CsPIPs* in response to drought, salt, and cold stress (Figure 10) and identified that *CsPIP1;1*, *CsPIP2*;1, *CsPIP2;3*, *CsPIP2;4*, and *CsPIP*2;10 might be closely related to tea plant stress responsiveness. Moreover, the H_2_O_2_ transport ability assay in yeast showed that CsPIP2;3 and CsPIP2;10 could transport H_2_O_2_ in the tea plant (Figure 10B), indicating their potential roles in the tea plant response to stress. Interestingly, stress-responsive *AQP* expression is further modulated by ABA, trehalose, and other signaling molecules. Particularly, we found that there are many ABA-related ci*s*-elements, including ARE and ABRE, identified in the promoters of *CsAQP*s. Although ABA treatment responses were not experimentally tested here, prior work confirms ABA-mediated regulation of several *CsAQPs*. These findings collectively indicate that ABA-dependent *CsAQPs* responses are critical for tea plant stress adaptation.

Additionally, tea plants exhibit high Al tolerance and are recognized as Al hyperaccumulators. AQPs have been validated to transport Al in plants. In this study, we analyzed the expression patterns of CsAQPs under Al treatment to identify candidates involved in Al transport. Results showed downregulation of most genes, while the expressions of *CsNIP4;1*, *CsTIP4;1*, *CsTIP4;2*, *CsPIP2;7*, and *CsSIP1;1* were upregulated. In *Arabidopsis thaliana*, the expression of *AtNIP1;2* is rapidly induced by Al stress, and loss-of-function mutants display increased Al sensitivity, indicating its role in Al uptake, translocation, and tolerance [56]. Notably, *AtNIP1;2* contains a WVAR signature in its Ar/R selectivity filter, identical to motifs in *CsNIP4;1* and *CsNIP4;2*. Previous studies also report induced *CsNIPs* transcription during Al treatments in the tea plant [57]. These upregulated *CsAQPs*, particularly *CsNIPs*, may contribute significantly to tea plant Al resistance.

It has been well known that AQPs control plant innate immunity via mediating H_2_O_2_ transport into the plant cell. Anthracnose and gray blight represent key fungal diseases in tea plants. Transcriptomic and proteomic studies have explored tea plant responses to these pathogens, yet key regulatory genes remain unidentified. Analysis revealed significant upregulation of several *CsPIPs, CsTIPs,* and *CsNIPs* genes during both infections under biotic stress. Crucially, the transcription levels of *CsPIP2;1*, *CsPIP2;3*, and *CsPIP2;10* increased during infections, and they were determined to facilitate H_2_O_2_ transport in tea plants (Figure 10B), indicating their crucial roles in tea plants’ pathogen response. This aligns with established PIP functions in biotic stress adaptation: rice *OsPIP2;2* mediates H_2_O_2_ transport to regulate immunity [54], while phosphorylated *TaPIP2;10* enables cytoplasmic H_2_O_2_ transport for enhanced defense in wheat [58]. Collectively, our findings present a systematic characterization of the *CsAQPs* family in the tea plant, establishing a critical foundation for future functional studies.

## 4. Materials and Methods

### 4.1. Identification of AQP Genes in Tea Plant

The whole genome of the tea plant was downloaded from the TPIA (http://tpia.teaplants.cn/ (accessed on 24 January 2024)). The protein sequences of AQPs in the *Arabidopsis thaliana* (http://www.arabidopsis.org/index.jsp (accessed on 28 February 2024)), *Vitis vinifera* (http://bioinformatics.psb.ugent.be/orcae/ (accessed on 4 April 2024)), *Vigna angularis* (https: //www.ncbi.nlm.nih.gov/genome/?term=vigna%20angularis (accessed on 11 April 2024)), were used as queries in this research. The protein sequences of 35 AQP genes identified in *Arabidopsis thaliana* were BLASTed against the entire protein sequence of the tea plant to obtain candidate *CsAQPs* gene protein sequences. Because XIP does not exist in *Arabidopsis thaliana*, we used *Vitis vinifera* XIPs for blast searching. After predicting conserved domains and removing genes that lacked these domains, 61 *CsAQPs* gene family members were identified. The physicochemical properties of the *CsAQPs* family members’ proteins were predicted using the ProtParam (https://web.expasy.org/protparam/ (accessed on 15 April 2024)) and ProtScale (https://web.expasy.org/protscale/ (accessed on 15 April 2024)) analysis tools. Transmembrane helices were predicted using DeepTMHMM-1.0 services (https://services.healthtech.dtu.dk/services/DeepTMHMM-1.0/ (accessed on 15 May 2024)).

### 4.2. Gene Structure, Motif Composition, and Domains Analysis in Tea Plant

The protein sequences of the CsAQPs gene family were analyzed using the MEME (https://meme-suite.org/meme/tools/meme (accessed on 20 April 2024)) to identify the conserved motifs. The gene family’s motifs were visualized using TBtools visualization module [59]. The conserved domains of the CsAQPs gene family members were exported using the Batch-CD-Search function of NCBI and visualized with TBtools.

### 4.3. Phylogenetic Tree Analysis of CsAQPs Gene Family Members

The *AQP* gene family sequences were extracted from the whole genomes of *Vitis vinifera*, *Arabidopsis thaliana*, and *Vigna angularis*. An unrooted phylogenetic tree was constructed using MEGA11 [60] with the neighbor-joining method based on the LG model, and 1000 bootstrap test replicates were used during the construction. The phylogenetic tree was illustrated using Evolview v3 (https://www.evolgenius.info/evolview/#/ (accessed on 16 December 2024)). The combined tree was generated to systematically classify AQPs, and the systematic names were assigned based on their evolutionary relationships.

### 4.4. Chromosome Localization and Gene Duplication Analysis of CsAQPs Gene Family Members

Synteny files were analyzed with default parameters using TBtools, and the synteny map of the tea plant was visualized using TBtools. The GFF3 file of the tea plant was analyzed and visualized using TBtools to determine chromosomal locations. The number of synonymous substitutions per synonymous site (Ka), the number of nonsynonymous substitutions per nonsynonymous site (Ks), and the *p*-value from Fisher’s exact test of neutrality were calculated using TBtools. A Ka/Ks ratio <1 indicates purifying selection, a Ka/Ks ratio = 1 indicates neutral selection, and a Ka/Ks ratio >1 indicates positive selection. The Ks value was calculated for the AQP homologous gene pairs and was used to calculate the gene duplication age T = Ks/2λ, λ = 6.5 × 10^−9^.

### 4.5. Prediction of Protein 3D Structures

Five CsAQPs protein sequences were submitted to the Phyre2 server (https://www.sbg.bio.ic.ac.uk/phyre2/html/page.cgi?id=index (accessed on 12 February 2025)) to predict the three-dimensional (3D) structures under ‘Normal’ mode based on homologous modeling. The CsPIP1;1 model was constructed based on the *Spinacia oleracea* PIP2;1 template, with a sequence identity of 72%. The CsTIP1;1 model was constructed based on the *Arabidopsis thaliana AtTIP2;1* template, with a sequence identity of 59%. The CsNIP1;1 model was constructed based on the *Oryza sativa* NIP2;1 template, with a sequence identity of 48%. The CsSIP2;1 model was constructed based on the *Homo sapiens* SIP2;1 template, with a sequence identity of 21%. Finally, the CsXIP2;1 model was also constructed based on the *Homo sapiens* Aquaporin-5, with a sequence identity of 28%. Subsequently, the predicted protein models and pore morphology were confirmed using the program in the PoreWalker. PoreWalker [61] (https://www.ebi.ac.uk/thornton-srv/software/PoreWalker/ (accessed on 12 February 2025)) with the protein database file (Protein Data Bank, PDB format) generated by Phyre 2. All line charts were generated using Origin 2025b.

### 4.6. Interaction Network Construction of CsAQPs

The STRING (http://string-db.org/cgi (accessed on 3 January 2025)) database was used to predict the interaction network of CsAQPs based on their orthologs in the model plant *Arabidopsis thaliana*. The minimum required interaction score was set to 0.70, and the maximum number of interactors was set to no more than 5.

### 4.7. Analysis of Regulatory Cis-Acting Elements

To further analyze the regulatory mechanisms of the *CsAQPs* genes in the tea plant in response to stress and growth and development, the sequence of the region 2000 bp upstream of the translation start site of the *CsAQPs* genes was extracted from the whole genomes of *Camellia sinensis* cv. Shuchazao and the putative cis-acting elements were identified through the PlantCARE program (https://bioinformatics.psb.ugent.be/webtools/plantcare/html/ (accessed on 29 May 2024)). The cis-acting elements involved in abiotic stress responses, biotic stress responses, tissue element, light response element, the circadian rhythm, cell cycle, and core promoter elements were summarized and analyzed.

### 4.8. Expression Pattern Analysis of CsAQPs Under Stresses Using Transcriptome Data

Six publicly available RNA sequencing (RNA-seq) datasets were downloaded from the Short Read Archive of the NCBI database for expression analysis in different tissues of the tea plant (project accession number: PRJEB39502), in response to drought stress (project accession number: PRJCA012395), in response to cold stress (project accession number: PRJNA387105), in response to salinity (project accession number: PRJEB11522), in response to Al treatment (project accession number: PRJNA517582), and in response to anthracnose (project accession number: PRJNA595772) and gray blight (project accession number: PRJNA564655). A heatmap was generated and visualized using TBtools software. The color scale shown with the heatmap represents the TPM counts, and the ratios were log2 transformed.

### 4.9. Quantitative Real-Time PCR (qRT-PCR) Analysis of the Expression Patterns of CsPIPs in Response to Abiotic Stresses

Healthy, uniformly growing one-year-old clonal tea plant seedlings, *Camellia sinensis* cv. Fudingdabaicha were used to perform drought, salt, heat, and cold stress treatments as described by our previous reports [62]. To initiate the drought and salt stresses, tea plants were cultured in a nutrient solution (Coolaber, NSP1030) for at least four weeks until the tea plants grew new roots. The hydroponic solution with 25% (*w*/*v*) polyethylene glycol (PEG) 6000 was used to carry out drought stress, and the samples at 0 h, 6 h, 24 h, and 72 h were collected for gene detection. The hydroponics solution with 200 mM NaCl was used to induce salt stress, and the samples at 0 h, 24 h, 48 h, and 72 h were collected for analysis. To initiate cold stress, the tea plants were pre-cultured in the growth chambers (temperature: 25 ± 2 °C, light/dark: 14/10 h, humidity: 75 ± 5%, light intensity: 200 mmol m^−2^ s^−1^) for four weeks. After that, tea plants were transferred into growth chambers with 4 °C for cold treatment, and the samples at 0 h, 6 h, 12 h, and 24 h were used for the analysis. For sampling, the top of third leaves were collected and immediately frozen in liquid nitrogen and stored at −80 °C until RNA extraction. Each treatment was carried out in triplicate.

For gene expression analysis, total RNA was extracted using an RNAprep Pure Plant Kit (Tiangen, Beijing, China), and qRT-PCR detection was conducted as previously described using an SYBR Green PCR Kit (Takara, Beijing, China) on a QuanStudio^TM^ 1 Real-Time PCR instrument [63]. The CsActin gene was used as the internal reference [64]. Gene-specific primers were designed using Primer Premier 6.0 software and listed in Table S4. Relative gene expression levels were calculated using the 2^−∆∆Ct^ method [65].

### 4.10. Transformation and Functional Analysis of CsPIP Genes in Yeast

To investigate the function and subcellular localization of *CsPIP* genes, these genes were transformed into yeast. The coding sequences of *CsPIP* genes were amplified using PrimeSTAR^®^ GXL DNA Polymerase (Takara, Beijing, China) and cloned into the vector pYES2-green fluorescent protein (GFP) using the In-Fusion^®^ Snap Assembly Master Mix (Takara, Beijing, China) using primers listed in Table S4. The constructed plasmids and the empty plasmid were then transformed into the yeast strain NMY51 by the lithium acetate method. The positive transformants were selected after culturing at 29 °C for 3 days on synthetic dropout medium-uracil (SD-URA) media with 2% (*w*/*v*) glucose. *CsPIP*-transformed yeast cell functional analysis and subcellular localization assays were conducted as previously described [66]. Briefly, yeast cells were incubated on SD-URA liquid medium at 29 °C for 24 h and then diluted with sterile water to OD 600 of 1.0, 1 × 10^−1^, 1 × 10^−2^, 1 × 10^−3^, 1 × 10^−4^ and 1 × 10^−5^ and then spotted on the SD-URA solid medium with 2% (*w*/*v*) galactose and 1% (*w*/*v*) raffinose with or without 3 mM H_2_O_2_, 800 mM mannitol, and 400 mM NaCl. The treated yeast cells were cultured at 29 °C for 3–4 days for analysis. To visualize the subcellular localization of CsPIPs in yeast, cells were directly aspirated and imaged through the GFP channel using an Olympus FV3000 laser confocal microscope.

## 5. Conclusions

In this study, 61 *CsAQPs* genes were identified from the tea plant genome. Their bioinformation characteristics, including encoded proteins, gene structures, chromosomal distributions, phylogenetic relationships, conserved motifs, and cis-acting elements, were comprehensively investigated. Like other plants, *CsAQPs* were classified into five subfamilies (PIP, TIP, NIP, SIP, and XIP). Expression profiling revealed tissue-specific and seasonal dynamics of *CsAQPs*, along with their responses to abiotic stresses (cold, drought, salinity), aluminum exposure, and biotic stresses (anthracnose, gray blight). Moreover, the potential functions of CsPIPs were investigated in yeast cells, and the key CsPIPs involved in H_2_O_2_ transport were identified. These findings highlight several candidate genes with significant regulatory roles under stress conditions. Overall, this work provides a foundational resource for elucidating the functional diversity of *CsAQPs* in the tea plant and prioritizes key targets for future mechanistic studies on their roles in stress adaptation and substance transport.

## Figures and Tables

**Table 1:** Characteristics of the 61 *CsAQP* genes identified from the tea plant genome.

No.	Gene_ID	AQP Names	Chromosome			Amino Acid Size	pI	MW (kD)	Gravy	Instability Index II	Aliphatic Index	Subcellular Location	TM
			Location	Start	End								
1	CSS0037800.1	*CsNIP1;1*	Chr8	136995561	137000663	282	7.99	29,702.39	0.518	23.92	102.7	plas, vacu	6
2	CSS0043800.1	*CsNIP1;2*	Contig505	39795	46791	284	7.87	30,201.05	0.462	25.98	101.69	plas	6
3	CSS0048617.1	*CsNIP1;3*	Chr8	79496692	79503950	284	7.87	30,219.08	0.452	27.63	100.32	plas	6
4	CSS0048884.1	*CsNIP4;1*	Chr1	178790789	178793605	273	8.60	29,473.41	0.62	29.48	107.03	plas	6
5	CSS0023412.1	*CsNIP4;2*	Chr1	178964297	178967104	273	8.30	29,461.33	0.592	30.01	104.18	plas	6
6	CSS0046969.1	*CsNIP4;3*	Chr10	56099626	56125155	270	7.49	28,857.92	0.607	33.01	103.56	plas	6
7	CSS0004550.1	*CsNIP4;4*	Chr10	56468721	56495248	270	8.05	28,884.98	0.595	33.01	103.56	plas	6
8	CSS0037780.1	*CsNIP4;5*	Contig505	250133	255868	487	6.72	52,713.64	0.287	42.96	95.89	plas	7
9	CSS0016100.1	*CsNIP4;6*	Chr8	79685216	79690890	430	6.72	46,338.14	0.261	41.18	92.95	plas	6
10	CSS0041534.1	*CsNIP5;1*	Contig1098	195986	203946	301	8.49	31,030.27	0.546	38.57	103.09	plas, vacu	6
11	CSS0037541.1	*CsNIP5;2*	Contig769	39569	47270	301	8.49	31,030.27	0.546	38.57	103.09	plas, vacu	6
12	CSS0044271.1	*CsNIP6;1*	Chr1	210183086	210191021	330	9.19	36,216.51	0.552	36.09	111.09	plas	7
13	CSS0046752.1	*CsNIP6;2*	Chr9	84773894	84774721	168	5.00	17,938.83	0.505	39.24	110.36	plas, vacu	4
14	CSS0019203.1	*CsNIP7;1*	Chr5	180407037	180407772	146	9.55	15,568.23	0.529	31.64	108.22	E.R	3
15	CSS0004643.1	*CsNIP7;2*	Chr5	180728972	180729707	146	9.55	15,568.23	0.529	31.64	108.22	E.R.	3
16	CSS0002350.1	*CsPIP1;1*	Chr7	141535384	141538493	287	9.07	30,661.75	0.409	35.37	96.55	plas	5
17	CSS0002218.1	*CsPIP1;2*	Contig911	164493	167653	287	9.07	30,661.75	0.409	35.37	96.55	plas	5
18	CSS0006638.1	*CsPIP1;3*	Chr12	148766960	148770897	286	8.84	30,799.9	0.404	30.75	94.55	plas	5
19	CSS0044037.1	*CsPIP2;1*	Chr4	170198609	170202694	286	8.65	30,690.69	0.457	31.93	97.59	plas	6
20	CSS0047143.1	*CsPIP2;2*	Chr10	112580306	112584212	284	7.85	30,066.93	0.541	33.77	105.53	plas	6
21	CSS0001968.1	*CsPIP2;3*	Chr3	2780552	2783224	280	9.07	29,812.79	0.467	37.53	93.4	plas	6
22	CSS0012183.1	*CsPIP2;4*	Chr1	20914959	20918006	287	8.29	30,655.69	0.518	36.25	102.02	plas	6
23	CSS0041392.1	*CsPIP2;5*	Chr5	98224028	98226368	231	8.87	24,579.65	0.615	38.67	103.07	plas, vacu, E.R	5
24	CSS0048879.1	*CsPIP2;6*	Chr6	172368952	172372365	280	9.73	29,855	0.476	32.78	99.68	plas	6
25	CSS0002360.1	*CsPIP2;7*	Chr7	121352829	121357062	282	8.56	30,207.15	0.512	35.14	103.57	plas	6
26	CSS0010462.1	*CsPIP2;8*	Chr2	47485491	47489338	267	9.69	28,497.67	0.626	31	105.28	plas, vacu	6
27	CSS0008573.1	*CsPIP2;9*	Chr2	52007967	52011541	267	9.69	28,497.67	0.626	31	105.28	plas, vacu	6
28	CSS0001305.1	*CsPIP2;10*	Chr2	47419505	47428276	280	9.44	30,015.04	0.418	31.11	97.96	plas	6
29	CSS0042042.1	*CsPIP2;11*	Chr2	52049352	52057255	280	9.47	29,989.01	0.411	31.38	97.96	plas	6
30	CSS0042437.1	*CsTIP1;1*	Chr4	167062250	167064557	258	6.96	27,175.69	0.755	26.87	111.63	plas, vacu	6
31	CSS0027319.1	*CsTIP1;2*	Chr13	67697561	67700534	252	5.89	26,244.41	0.734	25.02	107.66	plas, vacu, cyto	6
32	CSS0035495.1	*CsTIP1;3*	Chr10	103819178	103821244	251	6.38	25,984.1	0.74	23.83	105.82	plas, vacu	6
33	CSS0006794.1	*CsTIP1;4*	Chr11	62068430	62070604	235	5.02	24,186.17	0.872	34.12	110.94	plas, vacu	6
34	CSS0029404.1	*CsTIP1;5*	Chr7	136034768	136037454	252	5.02	26,232.37	0.784	24.21	106.11	plas, vacu	6
35	CSS0042658.1	*CsTIP1;6*	Chr1	26174103	26178326	252	5.48	25,967.18	0.837	20.59	112.38	plas, vacu	6
36	CSS0006277.1	*CsTIP1;7*	Chr4	146349836	146352066	252	4.78	26,047.1	0.789	27.97	104.21	vacu	6
37	CSS0005067.1	*CsTIP1;8*	Contig991	40413	42550	252	4.78	26,074.12	0.779	28.91	104.21	vacu	6
38	CSS0000601.1	*CsTIP2;1*	Chr15	20248925	20251370	248	6.21	25,272.39	0.886	24.43	111.33	vacu	6
39	CSS0042070.1	*CsTIP2;2*	Chr15	19947320	19949742	248	6.21	25,320.44	0.874	24.43	109.76	vacu	6
40	CSS0004021.1	*CsTIP2;3*	Chr12	137934609	137932052	212	9.24	22,855.77	0.627	50.29	109.06	plas	5
41	CSS0035641.1	*CsTIP3;1*	Chr15	117952879	117954487	265	6.92	27,880.37	0.559	38.88	106.87	plas, cyto_plas	6
42	CSS0019278.1	*CsTIP4;1*	Chr14	39586292	39587511	199	6.22	21,034.69	0.769	32.72	116.58	plas, vacu	5
43	CSS0011941.1	*CsTIP4;2*	Chr14	18021146	18022642	248	6.38	26,053.67	0.84	25.74	115.6	plas, vacu	6
44	CSS0042512.1	*CsTIP5;1*	Chr2	185747730	185748326	177	9.44	19,114.39	0.672	32.49	101.19	chlo	3
45	CSS0037352.1	*CsTIP5;2*	Chr10	76694846	76695538	201	8.63	21,347.13	0.679	34.45	101.29	plas, vacu	4
46	CSS0016664.1	*CsTIP5;3*	Chr7	157166190	157167580	248	7.27	25,501.78	0.796	31.16	106.98	plas, vacu	6
47	CSS0026581.1	*CsTIP5;4*	Chr7	157000556	157002074	248	7.23	25,453.74	0.805	28.26	106.61	plas, vacu, cyto_plas	6
48	CSS0011896.1	*CsTIP5;5*	Chr8	95434617	95439386	250	8.67	27,013.83	0.748	32	103.72	plas	7
49	CSS0035294.1	*CsTIP5;6*	Chr8	95768112	95768858	248	8.79	26,435.2	0.881	35.14	116.33	plas, vacu	7
50	CSS0024253.1	*CsTIP5;7*	Chr8	95769846	95789705	247	9.87	26,517.45	0.706	37.01	104.62	plas, vacu, cyto_plas, E.R	6
51	CSS0048247.1	*CsTIP5;8*	Chr10	76651106	76653236	289	8.88	30,599.85	0.654	28.07	105.92	plas, vacu	6
52	CSS0011252.1	*CsTIP5;9*	Chr10	76674041	76683483	241	6.78	25,937.41	0.683	36.4	105.15	plas, cyto_plas	4
53	CSS0043068.1	*CsTIP5;10*	Chr8	95440450	95444708	267	7.70	28,123.17	0.843	39.73	111.42	plas, vacu	6
54	CSS0016225.1	*CsTIP5;11*	Chr8	95794547	95795148	178	4.92	18,612.62	0.724	23.23	103.43	plas, vacu	4
55	CSS0008215.1	*CsTIP5;12*	Chr8	95447488	95470180	243	7.23	25,655.22	0.772	32.79	112.3	plas, vacu, cyto	3
56	CSS0017618.1	*CsXIP1;1*	Chr4	41698260	41697513	217	9.64	23,384.92	0.909	35.26	126.77	plas	6
57	CSS0031679.1	*CsXIP2;1*	Chr6	177021652	177024036	326	7.53	34,917.89	0.596	37.37	109.82	plas, E.R	7
58	CSS0035300.1	*CsXIP2;2*	Chr6	176928737	176929531	264	8.30	28,038.26	0.9	30.48	120.45	vacu	6
59	CSS0000861.1	*CsSIP1;1*	Chr10	165921949	165926334	197	9.41	20,978.78	0.77	35.09	109.04	plas, vacu	5
60	CSS0034662.1	*CsSIP2;1*	Chr10	143948080	143951838	246	10.44	26,904.9	0.44	22.15	102.68	plas, vacu	5
61	CSS0006527.1	*CsSIP2;2*	Chr10	143990231	143993354	246	10.41	26,862.86	0.434	23.97	102.28	plas, vacu	5

pI: theoretical isoelectric point; MW: molecular weight; Gravy: grand average of hydropathy; TM: transmembrane helix; plas: plasma membrane; vacu: vacuolar membrane; E.R.: endoplasmic reticulum; cyto: cytoplasm; chlo: chloroplast.

**Table 2:** Amino acid compositions of the NPA motifs, Ar/R selectivity filters, and Froger’s positions of CsAQPs.

Name	NPA Motifs		Ar/R Selectivity Filter				Froger’s Positions				
	LB	LE	H2	H5	LE1	LE2	P1	P2	P3	P4	P5
CsPIP1;1	NPA	NPA	F	H	T	R	Q	S	A	F	W
CsPIP1;2	NPA	NPA	F	H	T	R	Q	S	A	F	W
CsPIP1;3	NPA	NPA	F	H	T	R	Q	S	A	F	W
CsPIP2;1	NPA	NPA	F	H	T	R	Q	S	A	F	W
CsPIP2;2	NPA	NPA	F	H	T	R	Q	S	T	F	W
CsPIP2;3	NPA	NPA	F	H	T	R	M	S	A	F	W
CsPIP2;4	NPA	NPA	F	H	T	R	Q	S	A	F	W
CsPIP2;5	NPA	NPA	F	H	T	R	Q	S	A	F	W
CsPIP2;6	NPA	NPA	F	H	T	R	M	S	A	F	W
CsPIP2;7	NPA	NPA	F	H	T	R	Q	S	A	F	W
CsPIP2;8	NPA	NPA	F	H	T	R	M	S	A	F	W
CsPIP2;9	NPA	NPA	F	H	T	R	M	S	A	F	W
CsPIP2;10	NPA	NPA	F	H	T	R	M	S	A	F	W
CsPIP2;11	NPA	NPA	F	H	T	R	M	S	A	F	W
CsTIP1;1	NPA	NPA	H	I	A	V	T	S	A	Y	W
CsTIP1;2	NPA	NPA	H	I	A	V	T	S	A	Y	W
CsTIP1;3	NPA	NPA	H	I	A	V	T	S	A	Y	W
CsTIP1;4	NPA	NPA	H	I	A	V	T	S	A	Y	W
CsTIP1;5	NPA	NPA	H	I	A	V	T	S	A	Y	W
CsTIP1;6	NPA	NPA	H	I	G	V	T	S	A	Y	W
CsTIP1;7	NPA	NPA	H	I	A	V	T	S	A	Y	W
CsTIP1;8	NPA	NPA	H	I	A	V	T	S	A	Y	W
CsTIP2;1	NPA	NPA	H	I	G	R	T	S	A	Y	W
CsTIP2;2	NPA	NPA	H	I	G	R	T	S	A	Y	W
CsTIP2;3	NPA	-	H	-	G	R	T	T	-	C	W
CsTIP3;1	NPA	NPA	H	I	A	R	T	A	A	Y	W
CsTIP4;1	NPA	NPE	H	I	A	R	I	S	A	Y	W
CsTIP4;2	NPA	NPA	H	I	A	R	T	S	A	Y	W
CsTIP5;1	NPT	NQA	N	V	G	Y	T	S	A	Y	W
CsTIP5;2	NPS	NSA	N	V	E	Y	T	A	A	Y	W
CsTIP5;3	NPA	NPA	S	V	G	Y	T	S	A	Y	W
CsTIP5;4	NPA	NPA	S	V	G	Y	T	S	A	Y	W
CsTIP5;5	NPT	NAA	N	V	G	Y	T	S	A	Y	W
CsTIP5;6	NPV	NLT	N	V	G	Y	I	S	S	Y	W
CsTIP5;7	NPA	NPT	N	V	G	Y	T	S	A	Y	W
CsTIP5;8	NLA	NPT	N	V	G	Y	T	S	A	Y	W
CsTIP5;9	NPE	NPA	N	V	M	Y	T	S	A	Y	W
CsTIP5;10	NPA	NPA	N	I	G	Y	T	S	A	Y	L
CsTIP5;11	---	NSA	N	V	R	Y	T	S	A	Y	W
CsTIP5;12	NLA	NQA	N	V	R	Y	T	S	A	Y	W
CsNIP1;1	NPA	NPA	W	V	A	R	F	S	A	Y	L
CsNIP1;2	NPA	NPA	W	V	A	R	F	S	A	Y	I
CsNIP1;3	NPA	NPA	W	V	A	R	F	S	A	Y	I
CsNIP4;1	NPA	NPA	W	V	A	R	F	S	A	Y	I
CsNIP4;2	NPA	NPA	W	V	A	R	F	S	A	Y	I
CsNIP4;3	NPA	NPA	W	V	G	R	F	S	A	Y	V
CsNIP4;4	NPA	NPA	W	V	G	R	F	S	A	Y	V
CsNIP4;5	NPA	NPA	W	V	A	R	F	S	A	Y	I
CsNIP4;6	NPA	NPA	W	V	A	R	F	S	A	Y	I
CsNIP5;1	NPS	NPV	A	I	G	R	F	T	A	Y	L
CsNIP5;2	NPS	NPV	A	I	G	R	F	T	A	Y	L
CsNIP6;1	NLV	NLA	G	S	G	R	L	T	A	Y	V
CsNIP6;2	---	NPA	-	S	A	R	F	S	A	Y	V
CsNIP7;1	---	NPA	-	V	G	R	Y	S	A	Y	L
CsNIP7;2	---	NPA	-	V	G	R	Y	S	A	Y	L
CsSIP1;1	NPT	NPA	-	V	P	N	M	A	A	Y	W
CsSIP2;1	NPL	NPA	S	-	G	S	F	V	A	Y	W
CsSIP2;2	NPL	NPA	S	-	G	S	F	V	A	Y	W
CsXIP1;1	NPI	NPA	V	V	A	R	V	-	-	-	-
CsXIP2;1	NPI	NPA	I	V	A	R	V	C	A	F	W
CsXIP2;2	NPI	NPA	I	V	A	R	V	C	A	F	W

## Data Availability

Data is contained within the article and Supplementary Materials.

## Supplementary Materials

The following supporting information can be downloaded at: https://www.mdpi.com/article/10.3390/plants14243786/s1, Figure S1: Sequences alignment of CsAQPs; Figure S2: The sequence of 20 motifs; Figure S3. Protein interaction network of CsAQPs in the tea plant based on the STRING database. Table S1: Gene duplication event analysis of *CsAQPs*; Table S2: The Ka/Ks ratios of *CsAQPs* collinearity gene pairs in tea plant; Table S3: Analysis of cis-acting element in *CsAQPs* genes; Table S4: Primers used in this study.
