# Supplementary figures and images for: Genome-Wide Identification and Expression Analysis of Aquaporin Gene Family in Tea Plant (Camellia sinensis)
Source: Plants (Basel). 2025 Dec 12;14(24):3786. doi: 10.3390/plants14243786 (PMC12737062; doi:10.3390/plants14243786)

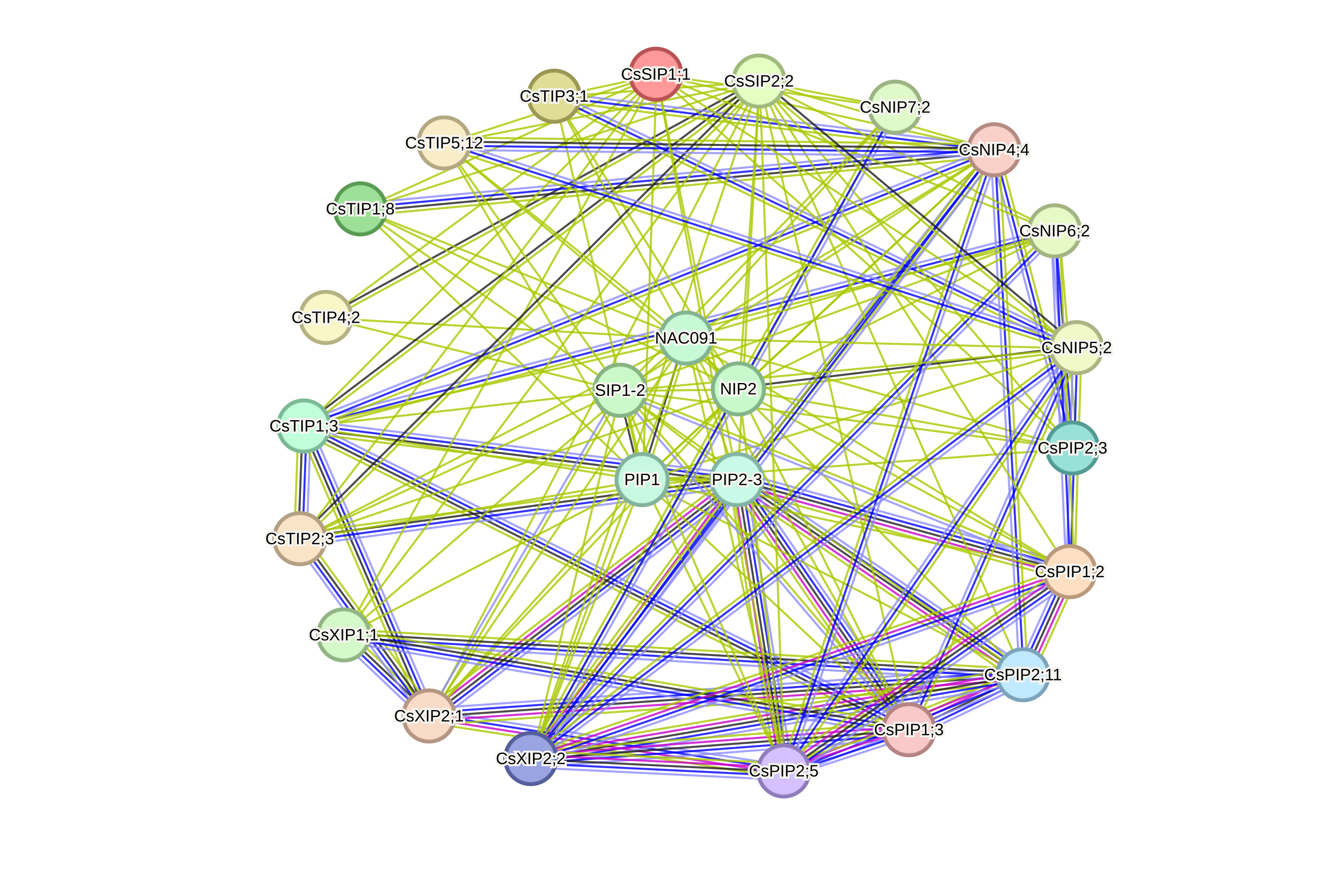

Supplement: Supplementary file 1 [file plants-14-03786-s001.zip › Figure S3.png]

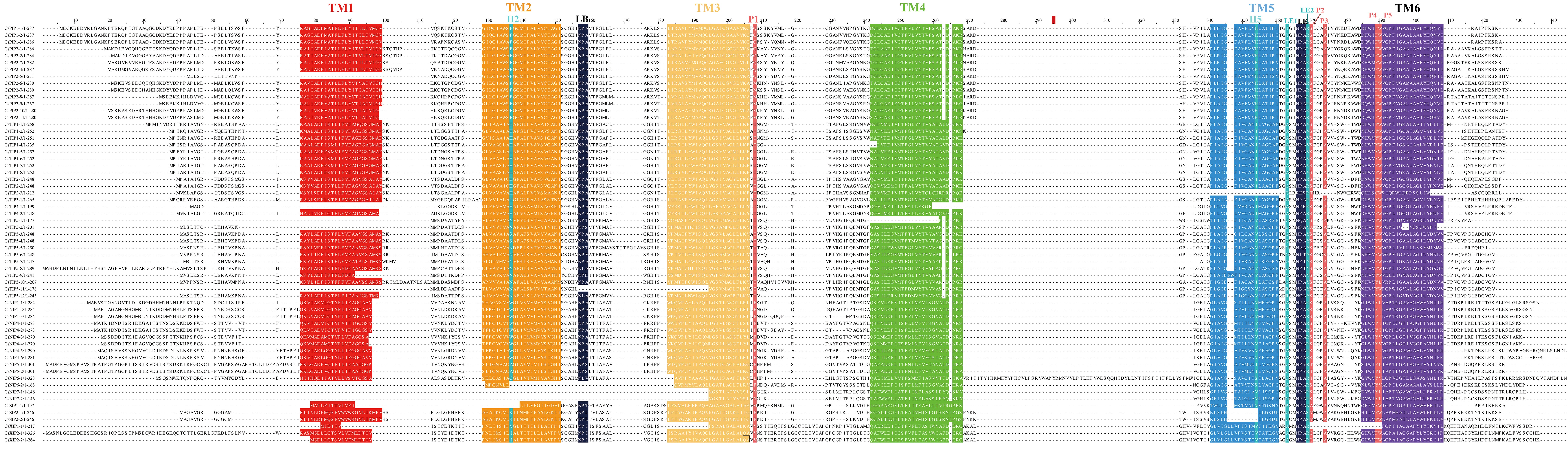

Supplement: Supplementary file 1 [file plants-14-03786-s001.zip › FigureS1 .jpg]
